# The impact of COVID-19 on liver transplantation programs in Austria

**DOI:** 10.1007/s00508-022-02105-z

**Published:** 2022-11-11

**Authors:** Lukas Hartl, Elisabeth Tatscher, Melanie Weiss, Lorenz Balcar, Robert Strassl, Mathias Jachs, Mattias Mandorfer, Thomas Soliman, Vanessa Stadlbauer, Peter Schemmer, Gabriela Berlakovich, Herbert Tilg, Stefan Schneeberger, Michael Trauner, Peter Fickert, Thomas Reiberger, Ivo Graziadei

**Affiliations:** 1 Division of Gastroenterology and Hepatology, Department of Medicine III, Medical University of Vienna, Waehringer Guertel 18–20, 1090 Vienna, Austria; 2 Vienna Hepatic Hemodynamic Lab, Division of Gastroenterology and Hepatology, Department of Medicine III, Medical University of Vienna, Vienna, Austria; 3 Division of Gastroenterology and Hepatology, Medical University Graz, Graz, Austria; 4 Division of Clinical Virology, Department of Laboratory Medicine, Medical University of Vienna, Vienna, Austria; 5 Division of Transplantation, Department of Surgery, Medical University of Vienna, Vienna, Austria; 6 General, Visceral and Transplant Surgery, Department of Surgery, Medical University of Graz, Graz, Austria; 7 Department of Internal Medicine I, Medical University of Innsbruck, Innsbruck, Austria; 8 Department of Visceral‑, Thoracic- and Transplantsurgery, Medical University of Innsbruck, Innsbruck, Austria; 9 Department of Internal Medicine, Academic Teaching Hospital Hall in Tirol, Milserstraße 10, 6060 Hall in Tirol, Austria

**Keywords:** SARS-CoV‑2, COVID-19, Liver cirrhosis, Liver transplantation, Vaccination

## Abstract

**Background:**

Coronavirus disease of 2019 (COVID-19) has affected liver disease management. The impact of the COVID-19 pandemic on the Austrian orthotopic liver transplantation (OLT) programs, however, has not been systematically investigated.

**Methods:**

All patients listed for OLT in Austria during 2020–2021 were studied. Data on severe acute respiratory syndrome coronavirus 2 (SARS-CoV-2) testing, vaccinations, infections, mortality and the overall number of OLTs (vs. pre-COVID-19: 2015–2019) were analyzed.

**Results:**

Overall, 490 patients (median age: 58.0 years, 70.4% men, hepatocellular carcinoma: 27.3%) were listed for OLT in Austria in 2020–2021. Alcohol-related cirrhosis (35.3%), cholestatic (16.7%) and viral liver disease (13.9%) were the main etiologies. Of the patients 61.2% underwent OLT and 8.8% died while on the waiting list.

The number of OLTs performed during COVID-19 (2020: *n* = 150; 2021: *n* = 150) remained unchanged compared to pre-COVID-19 (median: *n* = 152).

Among waiting list patients, 7.7% (*n* = 31/401) were diagnosed with COVID-19 and 7 (22.6%) of these patients died.

By the end of 2021, 45.1% (*n* = 176/390; 82.8% mRNA vaccinations) and 28.8% (105/365) of patients received 2 and 3 SARS-CoV‑2 vaccinations, respectively. After two SARS-CoV‑2 vaccinations, antibodies more often remained undetectable in patients vaccinated post-OLT (25.6% vs. 6.5% in patients vaccinated pre-OLT; *p* = 0.034). Patients with three vaccinations after OLT had lower antibody titers than patients vaccinated pre-OLT (post-OLT: 513.5, IQR 44.4–2500.0 vs. pre-OLT: 2500.0, IQR 1462.0–2500.0 BAU/mL; *p* = 0.020).

**Conclusion:**

The number of OLTs in Austria remained unchanged during COVID-19. SARS-CoV‑2 infections were rare but associated with high mortality in patients on the Austrian OLT waiting lists. SARS-CoV‑2 vaccination rates at the end of 2021 were suboptimal, while serological response was better in patients vaccinated pre-OLT vs. post-OLT.

## Introduction

The coronavirus disease of 2019 (COVID-19) pandemic, which originated in Wuhan, China in late 2019 [1, 2], causes considerable morbidity and mortality due to the continuous spread of severe acute respiratory syndrome coronavirus 2 (SARS-CoV-2) [3, 4]. COVID-19 reached Europe in early 2020, necessitating drastic measures to limit the spread of the virus [5]. These included the implementation of protective equipment, banning of large gatherings, travel restrictions, as well as physical distancing, also leading to a temporarily significant downscaling of in-hospital care for chronically ill patients, particularly in outpatient clinics and reduction of face-to-face visits [5–9].

Advanced chronic liver disease (ACLD) patients are susceptible to severe courses of COVID-19 and may experience hepatic decompensation during or after SARS-CoV‑2 infections [4, 10–15]. Moreover, some studies have also reported worse outcomes among patients after solid organ transplantation compared to the general public [16, 17]. Thus, international societies rapidly published consensus statements for management of ACLD patients and patients after orthotopic liver transplantation (OLT) during the COVID-19 pandemic [6, 7, 18], emphasizing the need for guideline-conform treatment, but also recommending a decrease of face-to-face visits and outsourcing of laboratory testing to local laboratories [6, 18].

Even in the early phase of the pandemic, grave effects of COVID-19 on OLT concerning utilization of healthcare resources, SARS-CoV‑2 infections of organ donors or recipients, increased OLT waiting list morbidity and increased liver-related mortality were expected [19–21].

Indeed, the OLT program was temporarily suspended by many transplant centers in the USA in early 2020 [22] and, organ availability significantly decreased in Italy [23]. In line, a global web-based survey found that the number of OLT candidates was significantly decreased early during the pandemic [24]. Later, the OLT frequency returned to normality and some centers even reported increased numbers of OLTs from deceased donors with a particular increase of alcoholic liver disease (ALD) as a reason for OLT during the pandemic [25–27].

Moreover, ACLD patients and particularly patients after OLT have poor antibody responses to SARS-CoV‑2 vaccines [28, 29], potentially leading to insufficient protection against COVID-19 in a significant number of patients.

The aims of this study were to investigate the impact of COVID-19 on OLT in Austria by assessing (i) the number of OLTs performed prior to and during COVID-19, (ii) the prevalence and outcome of SARS-CoV‑2 infections of patients on the OLT waiting list and (iii) the prevalence of SARS-CoV‑2 vaccination and antibody titer response among patients on the OLT waiting list during COVID-19.

## Material and methods

### Study population

This retrospective study included all patients on the OLT waiting list in the three Austrian OLT centers (Innsbruck, Vienna and Graz) during COVID-19 (i.e. 2020 and 2021). Clinical and laboratory parameters, including age, sex, date of listing for OLT, etiology of ACLD, presence of hepatocellular carcinoma (HCC), model for end-stage liver disease score (MELD) at the time of listing for OLT, SARS-CoV‑2 polymerase chain reaction (PCR) tests performed at the OLT center, SARS-CoV‑2 infection, SARS-CoV‑2 vaccination (2 or 3 times), type of SARS-CoV‑2 vaccine, SARS-CoV‑2 antibody titer response, OLT, death and COVID-19-related death were assessed by chart review. COVID-19-related death was defined as death due to respiratory, inflammatory, thromboembolic or hemodynamic complications after SARS-CoV‑2 infection. Notably, not all parameters were available for every patient due to the retrospective study design. Moreover, the number of OLTs performed in the years 2015–2019 (pre-COVID-19) was recorded.

### Laboratory parameters

All parameters were assessed by standard laboratory assays. Reverse transcriptase-PCR systems were used for SARS-CoV‑2 PCR testing [30]. For measurement of SARS-CoV‑2 antibody titers, the Elecsys Anti-SARS-CoV‑2 S immunoassay (Roche Diagnostics International Ltd, Rotkreuz, Switzerland) [31, 32] was used, quantifying antibodies to the receptor-binding domain of the viral spike protein of SARS-CoV‑2 with a detection range between 0.4 and 2500.0 binding antibody units per mL (BAU/mL). In line with previous studies [33], antibody concentrations of more than 0.8 BAU/mL were considered as positive SARS-CoV‑2 antibodies. Humoral immune response to vaccination was defined as presence of positive SARS-CoV‑2 spike protein-specific antibodies at least 4 weeks after the second or third standard vaccination.

### Statistical analysis

Number (*n*) and proportion (%) of patients exhibiting the parameter of interest were reported for categorical variables. Where appropriate, the total number of available values (total *n*) was added. Median with interquartile range (IQR) was used to report continuous data. To test for normal distribution, D’Agostino and Pearson and Shapiro-Wilk normality tests were implemented. Mann-Whitney U test was used for comparison of non-normally distributed continuous variables between two groups. Pearson’s χ^2^-testor Fisher’s exact test was used for group comparisons of categorical variables as appropriate. GraphPad Prism 8 (Graphpad Software, La Jolla, CA, USA) and IBM SPSS 25.0 statistic software (IBM, Armonk, NY, USA) were implemented for statistical analysis. A two-sided *p*-value of < 0.05 was considered statistically significant.

### Ethics

The study was approved by the ethics committees (EC) of the Medical University of Vienna, Graz and Innsbruck (EK 1461/2020; EK 1326/2021; EK 1336/2021). It was performed according to the current version of the Helsinki Declaration. Due to the retrospective study design, the need for informed consent was waived by the EC.

## Results

### Patient characteristics and outcomes (Table 1)

In total, 490 ACLD patients who were listed for OLT in Austria during the COVID-19 pandemic (i.e. in 2020 and 2021) were included in this study. The median age at listing for OLT was 58.0 years and 70.4% of patients were male. The most common ACLD etiologies were ALD (35.3%; *n* = 173), followed by cholestatic (16.7%; *n* = 82), viral liver disease (13.9%; *n* = 68) and non-alcoholic steatohepatitis (NASH; 8.2%; *n* = 40). 27.3% (*n* = 134) of patients had HCC. Median MELD at the time of listing for OLT was 15.0 points, 61.2% (*n* = 300) of ACLD patients listed for OLT during COVID-19 were transplanted until the end of 2021 and 8.8% (*n* = 43) died on the waiting list.

**Table 1:** Characteristics of patients listed for orthotopic liver transplantation (OLT) in Austria during COVID-19 (i.e. in 2020 and 2021)

Patient characteristics	Patients listed for OLT *n* = 490
*Sex, male/female (% male)*	345/145 (70.4)
*Age, years (IQR)*	58.0 (16.0)
*Main etiology of ACLD*	
ALD, *n* (%)	173 (35.3)
Viral, *n* (%)	68 (13.9)
NASH, *n* (%)	40 (8.2)
Cholestatic, *n* (%)	82 (16.7)
AIH	21 (4.3)
Cryptogenic, *n* (%)	23 (4.7)
Other, *n* (%)	83 (16.9)
MELD, median (IQR)^a^	15.0 (10.0)
Hepatocellular carcinoma, *n* (%)	134 (27.3)
Orthotopic liver transplantation, *n* (%)	300 (61.2)
Death on the waiting list, *n* (%)	43 (8.8)

*ACLD* advanced chronic liver disease; *AIH* autoimmune hepatitis; *ALD* alcoholic liver disease;* MELD* model for end-stage liver disease; *NASH* non-alcoholic steatohepatitis; *IQR* interquartile range

^a^At the time when the patient was listed for OLT

### Number of patients undergoing OLT in Austria prior to and during COVID-19 (Fig. 1*)*

**Fig. 1:**
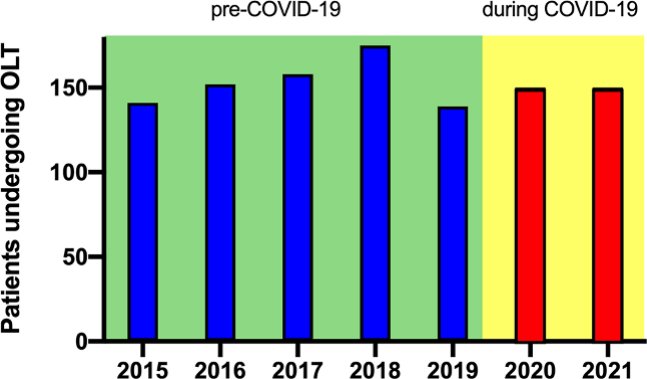
Number of patients undergoing orthotopic liver transplantation (OLT) in Austria prior to COVID-19 (i.e. 2015–2019) and during the COVID-19 pandemic (i.e. 2020–2021). Total number of patients undergoing OLT in Austria: 2015: *n* = 141; 2016: *n* = 152; 2017: *n* = 158; 2018: *n* = 175; 2019: *n* = 139; 2020: *n* = 150; 2021: *n* = 150

A total of 150 ACLD patients underwent OLT in 2020 and in 2021, which was approximately equal compared to the pre-COVID-19 period (2015–2019: median *n* = 152, IQR 26.5 patients), as detailed in Fig. 1. Interestingly, compared to the first half, 23.9% more patients were transplanted in the second half of 2020 (January–June/2020: *n* = 67 vs. July–December/2020: *n* = 83). Conversely, 38.1% more patients underwent OLT in the first half year of 2021 compared to the second half of the year (January–June/2021: *n* = 87 vs. July–December/2021: *n* = 63).

### Prevalence of SARS-CoV-2 PCR testing, SARS-CoV-2 infections and COVID-19-related deaths among patients on the OLT list during COVID-19 (Table 2; Fig. 2)

Of the ACLD patients listed for OLT during COVID-19, 81.8% (*n* = 401/490) were tested for SARS-CoV‑2 by PCR test at the OLT centers at least once, 7.7% (*n* = 31) of patients with available SARS-CoV‑2 PCR test were infected with SARS-CoV‑2, 7 patients with SARS-CoV‑2 infection died and all of these deaths were COVID-19-related. Thus, 1.4% of ACLD patients listed for OLT during COVID-19 had COVID-19-related death, which was equivalent to 22.6% of ACLD patient listed for OLT with SARS-CoV‑2 infection.

**Table 2:** Prevalence of SARS-CoV‑2 infections and outcomes of patients listed for orthotopic liver transplantation (OLT) in Austria during COVID-19 (i.e. in 2020 and 2021)

Parameters	Patients listed for OLT *n* = 490
SARS-CoV‑2 PCR available, *n* (%)	401 (81.8)
SARS-CoV‑2 positive, *n*/total *n* (%)	31/401 (7.7)
COVID-19-related death, *n*/total *n* (%)	7/31 (22.6)

*COVID-19* coronavirus disease of 2019; *PCR* polymerase chain reaction; *SARS-CoV‑2* severe acute respiratory syndrome-coronavirus‑2

**Fig. 2:**
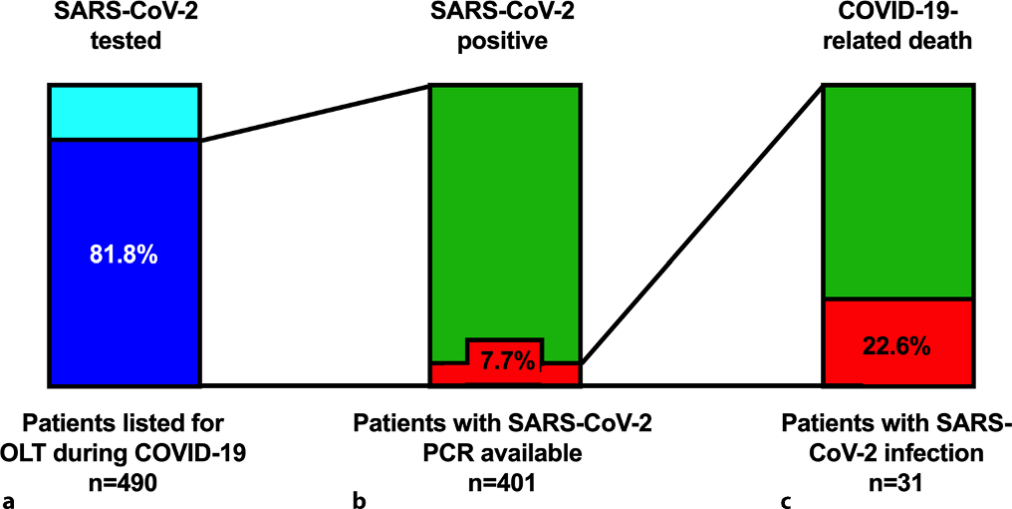
Proportion of SARS-CoV‑2 PCR testing, SARS-CoV‑2 infections, and COVID-19-related death on the liver transplantation list. **a** Proportion of patients listed for orthotopic liver transplantation (OLT) during COVID-19 (i.e. in 2020 and 2021), who were tested for SARS-CoV-2 at the OLT center (*blue*). **b** Proportion of SARS-CoV‑2 infections (red) among the patients tested during COVID-19. **c** Rate of COVID-19-related death (*red*) among SARS-CoV‑2 positive patients on the OLT list

### Prevalence of SARS-CoV-2 vaccination among patients on the OLT list during COVID-19 (Table 3; Fig. 3)

Of all patients with available information concerning SARS-CoV‑2 vaccination (*n* = 390/490), 45.1% (*n* = 176/390) were vaccinated at least twice at the end of 2021 and 82.8% (*n* = 125) of ACLD patients listed for OLT with at least 2 SARS-CoV‑2 vaccinations received an mRNA vaccination.

**Table 3:** Prevalence of vaccination and SARS-CoV‑2 titer response in patients listed for orthotopic liver transplantation (OLT) in Austria during COVID-19 (i.e. in 2020 and 2021)

Parameters	Number of patients listed for OLT	
*2* *×* *SARS-CoV‑2 vaccinated, n/Total n (%)*^*1*^	176/390 (45.1)	
*mRNA vaccination, n/Total n (%)*^*2*^	125/151 (82.8)	
*SARS-CoV‑2 antibody titer assessed, n/Total n (%)*	70/176 (39.8)	
*SARS-CoV‑2 antibody titer after 2 vaccinations*		
Vaccinated prior to OLT (*n* = 31), BAU/mL (IQR)	575.0 (2359.0)	*p* = 0.630
Vaccinated after OLT (*n* = 39), BAU/mL (IQR)	350.0 (2499.5)	
*SARS-CoV‑2 antibody titer not detectable after 2 vaccinations*		
Vaccinated prior to OLT, *n*/Total *n* (%)	2 (6.5)	*p* = 0.034
Vaccinated after OLT, *n*/Total *n* (%)	10 (25.6)	
*3* *×* *SARS-CoV‑2 vaccinated, n/Total n (%)*^*3*^	105/365 (28.8)	
*SARS-CoV‑2 antibody titer assessed, n/Total n (%)*	46/105 (43.8)	
*SARS-CoV‑2 antibody titer after 3 vaccinations*		
Vaccinated prior to OLT (*n* = 28), BAU/mL (IQR)	2500.0 (1038.0)	*p* = 0.020
Vaccinated after OLT (*n* = 18), BAU/mL (IQR)	513.5 (2455.6)	
*SARS-CoV‑2 antibody titer not detectable after 3 vaccinations*		
Vaccinated prior to OLT, *n*/Total *n* (%)	0 (0.0)	*p* = 0.207
Vaccinated after OLT, *n*/Total *n* (%)	1 (5.6)	

*SARS-CoV‑2* severe acute respiratory syndrome-coronavirus‑2

^a^number of patients with available information on at least 2 previous SARS-CoV‑2 vaccinations: *n* = 390/490 (79.6%)

^b^number of patients with available information on the type of received vaccination (mRNA vs. vector): *n* = 151/176 (85.8%)

^c^number of patients with available information on 3 previous SARS-CoV‑2 vaccinations: *n* = 365/490 (74.5%)

**Fig. 3:**
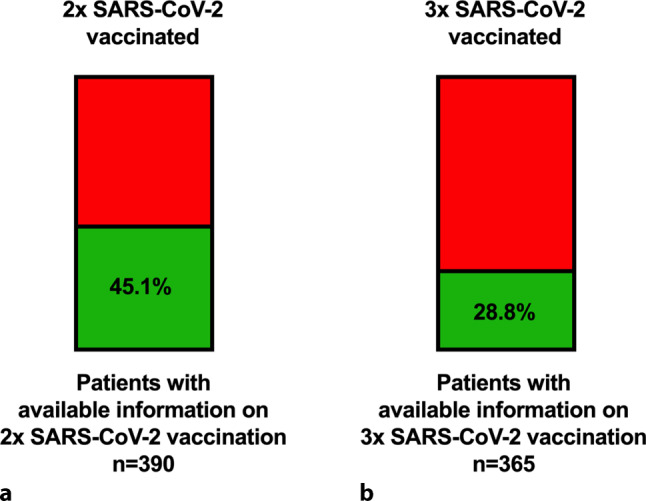
SARS-CoV‑2 vaccination rate on the Austrian OLT list. **a** Proportion of patients listed for liver transplantation (OLT) during COVID-19 (i.e. in 2020 and 2021) who had received at least two SARS-CoV‑2 vaccinations at the end of 2021. **b** Proportion of OLT-listed patients during COVID-19 who had received 3 SARS-CoV‑2 vaccinations at the end of 2021

Information on presence of three SARS-CoV‑2 vaccinations was available in 365 patients, 28.8% (*n* = 105) of these patients were 3 times SARS-CoV‑2 vaccinated, which was equivalent to 59.7% of patients with at least 2 SARS-CoV‑2 vaccinations.

### SARS-CoV-2 antibody response to vaccination in patients on the OLT list during COVID-19 (Table 3; Fig. 4 )

Out of all ACLD patients listed for OLT during COVID-19 with at least two SARS-CoV‑2 vaccinations, 39.8% (*n* = 70) had SARS-CoV‑2 titers determined after the second SARS-CoV‑2 vaccination. From these patients, 31 (44.3%) were vaccinated before OLT (pre-OLT) and 39 (55.7%) were vaccinated after OLT (post-OLT).

**Fig. 4:**
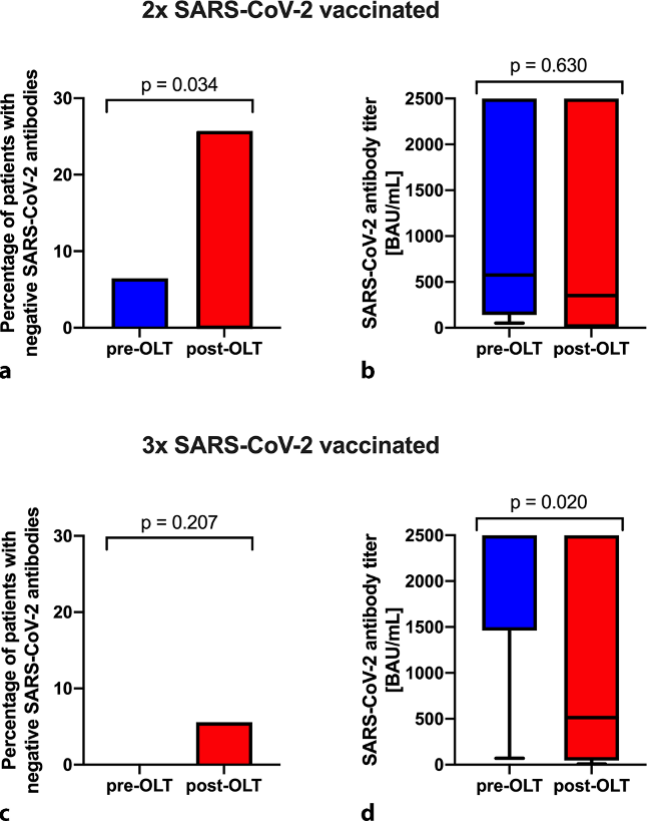
Response to SARS-CoV‑2 vaccinations in patients vaccinated pre-OLT and post-OLT. Comparison of (**a**) percentage of patients with insufficient SARS-CoV‑2 vaccination response (i.e. SARS-CoV‑2 antibody titer below the presumed threshold for protection) and **b** SARS-CoV‑2 antibody titers of patients, who had received two SARS-CoV‑2 vaccinations before (pre-OLT) and after orthotopic liver transplantation (post-OLT). **c** Comparison of percentage of patients with insufficient SARS-CoV‑2 antibody response and **d** SARS-CoV‑2 antibody titers of patients, who had received 3 SARS-CoV‑2 vaccinations pre-OLT and post-OLT

Overall, patients after three vaccinations had significantly higher SARS-CoV‑2 antibody titers (two vaccinations: 460.5 [IQR 53.7–2500.0]BAU/mL vs. three vaccinations: 2500.0 [IQR 227.0–2500.0]BAU/mL; *p* = 0.001).

Median SARS-CoV‑2 antibody titers were numerically lower among patients being vaccinated after OLT (pre-OLT: 575.0 [IQR 141.0–2500.0]BAU/mL vs. post-OLT: 350.0 [IQR 0.5–2500.0]BAU/mL), however, there was no statistically significant difference (*p* = 0.630), possibly due to the low sample size. While there were patients with undetectable SARS-CoV‑2 antibodies among both groups, either vaccinated pre-OLT (*n* = 2; 6.5%) or post-OLT (*n* = 10; 25.6%), the prevalence of patients with undetectable SARS-CoV‑2 antibodies was higher in the post-OLT SARS-CoV‑2 vaccination cohort (*p* = 0.034).

Among patients with three SARS-CoV‑2 vaccinations, 43.8% (*n* = 46) had SARS-CoV‑2 antibody titers available. Antibody titers were significantly higher in patients vaccinated pre-OLT (pre-OLT: 2500.0 [IQR 1462.0–2500.0]BAU/mL vs. post-OLT: 513.5 [IQR 44.4–2500.0]BAU/mL; *p* = 0.020) and there was only one patient with three vaccinations post-OLT with negative SARS-CoV‑2 antibody titer (*n* = 1/18; 5.6%).

## Discussion

In this large retrospective study, we thoroughly investigated the impact of the COVID-19 pandemic on the Austrian OLT scene in 2020 and 2021. We showed that despite an initial drop in OLTs, the overall number of OLTs were virtually unchanged compared to pre-COVID-19 throughout the COVID-19 pandemic. Although the prevalence of SARS-CoV‑2 infections was low among ACLD patients listed for OLT during COVID-19, the mortality of SARS-CoV‑2 infected patients was considerable (22.6%). At the end of 2021, vaccination coverage was poor among ACLD patients listed for OLT with only 45.1% of patients being SARS-CoV‑2 vaccinated at least twice. Importantly, patients who were vaccinated pre-OLT had higher SARS-CoV‑2 antibody titers and lower prevalence of negative SARS-CoV‑2 antibodies after vaccination.

The COVID-19 pandemic impacted on patients undergoing OLT and in the postoperative phase in numerous aspects. Firstly, worldwide transplant centers reported decreased number of OLTs performed particularly during the initial stage of COVID-19 [22–24], followed by an increase in the number of OLTs [25–27]. In line with these findings, the number of OLTs was relatively lower during the first half year of the pandemic with a subsequent increase in OLTs, particularly during the second half year of 2020 and the first half year of 2021; however, the overall number of patients undergoing OLT in Austria during COVID-19 (2020 and 2021) was unchanged when compared to pre-COVID-19 (i.e. 2015–2019). Interestingly, the number of deceased liver donors increased in 2020–2021 in the Eurotransplant region, unlike the number of other organ donors (e.g. heart and lung donors, which decreased during this time period) [34]. Moreover, in contrast to some other European OLT centers [35, 36], the OLT program in Austria was never completely shut down during the pandemic.

While the majority of ACLD patients listed for OLT during COVID-19 underwent PCR testing for SARS-CoV‑2, the prevalence of SARS-CoV‑2 infections was low in this population (7.7% of patients tested). We can only speculate about the reasons for this fact; however, national and international societies published recommendations at the beginning and also throughout the pandemic [6, 7, 18, 37], emphasizing the need for reduction of face-to-face contacts and social distancing for ACLD patients and patients after OLT. Thus, it might be the case that ACLD patients on the OLT list were well-informed about the high risk of SARS-CoV‑2 infections [10, 15] and may have been particularly restrictive and careful with social interactions.

Among ACLD patients listed for OLT with SARS-CoV‑2 infection, mortality was very high, since almost one quarter deceased. Notably, all these deaths were COVID-19-related. This is in line with previously reported high mortality of SARS-CoV‑2 infection in ACLD patients [10, 14, 15].

Safe and efficient vaccines for SARS-CoV‑2 have been available since the end of 2020 [38–40], which was emphasized by a statement issued by the Austrian Society for Gastroenterology and Hepatology (ÖGGH) in February 2021 that also recommended SARS-CoV‑2 vaccination to all stable ACLD patients and patients after OLT [41]. Nevertheless, SARS-CoV‑2 vaccination rates were poor at the end of 2021, with only 45.1% of ACLD included patients being vaccinated at least twice and 28.8% of patients being vaccinated three times, despite information campaigns in all three centers. This might be due to high morbidity among ACLD patients on the OLT list; however, we cannot rule out that skepticism and concerns towards the vaccines may have played a role in this, as well.

In line with previous literature, antibody titer response was negative in ACLD patients and especially in patients post-OLT after two SARS-CoV‑2 vaccinations [28, 29]. Fittingly, SARS-CoV‑2 antibody titers after three vaccinations were higher in patients, who were vaccinated pre-OLT. One patient, who was vaccinated post-LT had negative SARS-CoV‑2 antibodies even after three vaccinations. This emphasizes the importance of SARS-CoV‑2 vaccination before OLT in order to be reliably sufficiently protected against severe courses of COVID-19.

Our study also has limitations: firstly, because of the retrospective study design, selection bias cannot be completely ruled out; however, we tried to minimize selection bias by including all patients who were listed for OLT during COVID-19. Moreover, not all variables were available for every single patient, which holds especially true for information on (three time) vaccination and antibody titer response. While this certainly is a limitation of the study, our results are well in line with previous publications and represent a real-life situation. Furthermore, the number of patients who received a single dose of SARS-CoV‑2 vaccine was not evaluated. Moreover, as we did not assess etiology of liver disease in patients undergoing OLT pre-COVID-19, we cannot analyze, whether there was a change in liver disease etiology in patients undergoing OLT during COVID-19; however, an increase in ALD during the pandemic has been reported in other studies [25–27] and progressive cholestasis, as well as secondary sclerosing cholangitis after COVID-19 are also relevant for OLT during COVID-19 [14].

In conclusion, we were able to show that the COVID-19 pandemic had diverse effects on ACLD patients on the OLT list, as well as on patients after OLT in Austria. Overall, the numbers of OLTs performed in Austria were stable compared to pre-COVID-19 (i.e. 2019); however, new challenges, including the need for testing for SARS-CoV‑2, arose. The prevalence of SARS-CoV‑2 infections of patients on the OLT list was low but linked to considerable mortality. At the end of 2021, less than half of ACLD patients on the OLT list during COVID-19 were SARS-CoV‑2 vaccinated at least twice and less than one third were vaccinated three times. Assessed by SARS-CoV‑2 antibody titers, patients who were vaccinated pre-OLT responded better with lower rates of negative SARS-CoV‑2 antibodies.

## Abbreviations

ACLD: Advanced chronic liver disease
ALD: Alcoholic liver disease
BAU/mL: Binding Antibody Units/milliliter
COVID-19: Coronavirus disease 2019
EC: Ethics committee
HCC: Hepatocellular carcinoma
IQR: Interquartile range
MELD: Model for end-stage liver disease
MRNA: Messenger ribonucleic acid
NASH: Non-alcoholic steatohepatitis
OLT: Orthotopic liver transplantation
PCR: Polymerase chain reaction
SARS-CoV‑2: Severe acute respiratory distress syndrome coronavirus 2
